# Giving patients a voice for healthcare reform in Austria: the qualitative voice-study

**DOI:** 10.1093/fampra/cmae031

**Published:** 2024-05-25

**Authors:** Kathryn Hoffmann, Silvia Wojczewski, Nicole Rumpler, Aaron George, Pauline de Boeckxstaens

**Affiliations:** Department for Primary Care Medicine, Center for Public Health, Medical University of Vienna, Vienna 1090, Austria; Department for Primary Care Medicine, Center for Public Health, Medical University of Vienna, Vienna 1090, Austria; Department for Primary Care Medicine, Center for Public Health, Medical University of Vienna, Vienna 1090, Austria; Meritus Health, Hagerstown, MD 21742, United States; Department of Public Health and Primary Care, Ghent University Faculty of Medicine and Health Sciences, Ghent, 9000, Belgium

**Keywords:** primary care, healthcare service, health system planning, patient inclusion, healthcare reform, patient participation

## Abstract

**Background:**

Inclusion of patients in healthcare service and system planning is an increasingly important tool to improve healthcare systems worldwide. In 2012, a focused healthcare reform was initiated in Austria to strengthen the primary care sector which is still underway in 2023.

**Objective:**

The aim of this study was to assess the perceptions, desires, and needs of patients in terms of primary care as a necessary building block of the Austrian healthcare reform.

**Methods:**

This study was designed as an exploratory qualitative study using semi-structured interviews between the years 2013 and 2018. Interviews with patients focused on positive and negative experiences with regard to general practice (GP) consultations and perceptions of the primary care system in general, as well as desires for improvement. Qualitative content analysis was used to analyse the material using the software atlas.ti.

**Results:**

Altogether, 41 interviews were conducted with seven categories identified. These categories include organization and time management around consultation, access, and availability including opening hours, human and professional aspects of consultation, infrastructure and hygiene of the waiting room, healthcare system factors, as well as non-clinical/administrative staff.

**Conclusions:**

Appreciating and responding to patients’ perceptions and needs, healthcare reform in Austria should include improvements regarding consultation/waiting time, coordination, and navigation in Primary Care. Successful healthcare reform has to include the patient voice.

**Key messages**

- Inclusion of patients’ voices is crucial for improving healthcare services.
- This is the first study in Austria on patient’s desires for primary care.
- Patients wish for improvements regarding waiting time and coordination.
- Patients’ voices should be integrated on a regular basis for healthcare reform.

## Introduction

Inclusion of patients in healthcare service and system planning is an increasingly important tool to improve healthcare systems worldwide [1–4]. Decades of research support that patient satisfaction is tied to availability, accessibility, accommodation, acceptability, and affordability of care [5, 6]. In the UK, example.g. the involvement of patients is central to the promotion of continuous improvement in quality of care delivery [7]. Bochel *et al*. stated in their review that *“participation might be intended to improve governance, democracy, social capital, education and development of individuals, policies, service implementation and delivery* [1]*.”* In terms of healthcare planning and development, this suggests that patient involvement could lead to improved development of services, structures, and outcomes of care [2, 3].

In 2012, Kringos *et al*. published a revealing document on the strength of primary care in Europe, and within this Austria was identified with relatively underdeveloped primary care [8, 9]. Austria was found to have weaknesses in the governance of primary care (PC), workforce development, and coordination and continuity of care [8, 9]. Historically, the public Austrian healthcare system follows a Bismarck system which means universal health care where patients pay a fee to a social insurance fund that pays for treatments and it has been structured with only two levels of care, primary (general practice/primary care centres/mobile care services) and secondary care (medical specialists and hospitals). This is different from models elsewhere which typically involve three levels of care (primary, secondary, and tertiary level) [10]. Additionally, Austria has no clear demarcation line for access between primary and secondary care [10]. This organization is a barrier to the continuity and coordination role of primary care: Individuals can access with few exceptions secondary specialist care in the ambulatory sector without the knowledge or facilitation of a GP, and they do so in much higher numbers than the rest of Europe [10, 11]. Strengthening the primary care sector would probably lead to less specialist consultations—especially those consultations that could be performed by the primary care sector. In addition, mainly solo GP practices exist and primary care centres (*Primärversorgungszentren—PVE*) across the country remain low [12]. Consequently, a healthcare reform has been initiated in Austria, with the aim to strengthen the primary care sector.

In a first step, Austrian primary care reform aims to facilitate and increase the number of GP group practices with primary care teams, expand opening hours, and enhance service profiles either via centres or via networks [12]. This approach aims to make primary care practices more comprehensive and attractive for patients to access them as an initial point of care without having to add a clear demarcation line between primary and secondary care on a structural level. The first primary care centre was established in 2015 to encourage such access. As of 2023, with reform ongoing, there are now 24 primary care centres and networks established in Austria [13]. In these primary care centres there is usually a team of 2–3 GPs working together with other primary care professionals such as dieticians or nurses, and more rarely social workers, psychotherapists, or physiotherapists.

Alongside system and infrastructure development, it is essential to involve the voices of those receiving care. Qualitative studies on patient participation in Austria’s primary healthcare system are missing so far. Given this imperative, the aim of this study was to understand what patients value and identify for improvement in their perception of the existing publicly funded primary care setting by collection and analysis of people’s experience of GP consultations.

## Methods

The voice-study aimed to research the experiences and expectations of patients with general practice (GP) as the cornerstone of primary health care in Austria. The study was designed as an exploratory qualitative study using semi-structured interviews in accordance with the COREQ checklist (see Supplement 1). As qualitative research aims to gain deeper insight into a topic and understand the perception of people beyond prepopulated questionnaires, it is an ideal method for research in areas where understanding of local context and perception is limited.

### Recruitment

The recruitment of participants took place in the three most populous counties of Austria, namely Vienna, Lower Austria, and Upper Austria, via the snowball system. Vienna is the capital and is additionally a county as well as the only metropolis in Austria. Lower Austria is a more rural county and Upper Austria is mixed with industrial sites and rural areas. Four medical students used identical recruitment techniques and interview guidelines to conduct the interviews after being trained in qualitative interview techniques [14–17]. In Vienna, one student recruited German-speaking persons and the other Turkish-speaking persons. Turkey is the fourth largest migration group in Austria after Germany, Romania, and Serbia, and the second largest non-EU migration group after Serbia [18]. For the snowball system, each student recruited two persons within their circle of acquaintances. The participants were approached by telephone call or in a private meeting. No one declined to participate. After finishing the interviews, these participants recommended two new persons in a consistent manner until data saturation was reached for each county. Data saturation was agreed upon when more interviews were considered not to add any new insights to the main topic [19]. The aim was to recruit equal numbers of women and men, as well as equal persons from five age groups, namely 18–30, 31–45, 46–60, 61–75, and 76 years and older as well as including different educational backgrounds (primary, secondary, and tertiary education completed). Other inclusion criteria were compos mentis, living in the county for a minimum of five years, and having visited a publicly funded GP within the county at least once. All participants were informed about the study prior to their participation and had to sign an informed consent if they were willing to participate. None of the interview partners dropped out during the interview.

### Data collection

The interviews took place between 2013 and 2018. The participants were given the option to interview in an office at the Medical University of Vienna or at their homes. The setting for the interviews was a one-on-one between the interviewer and the interviewee. Before the start of the interview, the interviewees stated their sex, age group, and highest educational level in a short questionnaire, which was linked to the interview via a specific code. Semi-structured interviews were conducted for all participants. The interviews took between 30 and 90 minutes, with a mean of 50 min, and were digitally recorded. None of the interviews needed to be repeated. The interviews took place in the presence of the interviewee and interviewer only (see Table 1 for Interview Guideline).

**Table 1.** Interview guideline—English translation.

The interview guidelines for the semi-structured interviews contained five questions on their experiences, needs, and desires for improving their primary care experience. 1. Tell me about your last GP consultation at a public GP practice 2. What do you think about primary care in your county in general? 3. What do you like regarding your healthcare provided by GPs and GP practices? Why? Do you have examples? 4. What do you not like regarding your healthcare provided by GPs and GP practices? Why? Do you have examples? 5. If you would have the opportunity, what would you like to change and how?

Additional questions were raised on satisfaction and utilization of healthcare services if participants were not immediately responsive [5, 6].

### Data analysis

All digitally recorded interviews were transcribed, with the Turkish transcripts additionally translated into German by the interviewer who did the Turkish interviews. Qualitative content analysis was performed using the software *atlas.ti* [20]. The third author (NR) who was not involved in the interviews analysed all original transcripts. An inductive procedure was chosen by NR to analyse the transcripts in which codes were formed according to the *“language of the material”* [21]. Then, codes were also assigned deductively based on the themes of the interview guidelines. The first author (K.H.) coded at least 30% additionally, codes were compared and discussed by the two authors to check for coder reliability. K.H. and N.R. discussed their codes and merged the codes into broader categories. No major differences came up in the coding schemes of the two. For the purpose of this publication, the quotes were translated from German into English by the authors N.R., S.W., and K.H.

### Ethical considerations

Before the interviews, all participants were informed about the study and its goals. They had to sign a written consent form where they were informed that they could drop out of the study at any given moment with no negative consequences for them and that their data would then be deleted. This study has a positive ethical approval by the ethics committee of the Medical University of Vienna (Nr. 224/2019) and was conducted under European law regarding data protection and security.

## Results

A total of 41 interviews could be conducted in the three most populated regions in Austria: Vienna, Upper, and Lower Austria. Table 2 shows the demographics of the participants.

**Table 2.** Demographics of the participants.

Variable	Sub-variable	*n*	%
Overall		41	100
Sex	Female	18	43.9
	Male	23	56.1
Age group	18–30 years	10	24.4
	31–45 years	7	17.1
	46–60 years	13	31.7
	61–75 years	7	17.1
	76 years and older	4	9.8
Highest completed educational level	Primary	8	19.5
	Secondary	20	48.8
	Tertiary	13	31.7
County	Vienna—German speaking	10	24.4
	Vienna—Turkish speaking	8	19.5
	Lower Austria	12	26.8
	Upper Austria	11	29.3

Overall, 39 codes could be defined from the interview transcripts that we summarized under seven main categories: organization and time management around the consultations; aspects of access, availability, and opening hours of GP practices; professional aspects of consultation; human aspects of consultation; infrastructure and hygiene of the GP practice; overarching healthcare system aspects and last but not least aspects regarding the non-clinical/administrative staff. Table 3 gives an overview of the seven main themes and related subthemes (codes).

**Table 3.** Themes and subthemes.

Themes
**1. Organization/time management around the consultations** Subthemes: waiting time and circumstances prior to consultation; different options of appointment booking; duration of the consultation/consultation length; number of patients in the waiting room
**2. Access, availability, and opening hours** Subthemes: opening-hours; availability of GP services in the vicinity; home visits; accessibility; consultations via telemedicine; emergency service
**3. Consultation (professional aspects)** Subthemes: professional communication skills; coordination and navigation function; up to date diagnostic and therapeutic skills; broadening of services or additional services; prescription of medications; up to date medical education
**4. Consultation (human aspects)** Subthemes: established relationship; empathy and being taken seriously; interest in the patients living conditions; caring behaviour of the GP; stress of the GP; trust and reliability
**5. Infrastructure and hygiene of the GP practice** Subthemes: waiting room; technical infrastructure; special waiting room/area for children with parents; hygiene, air circulation and light; privacy at the reception desk
**6. Health system factors** Subthemes: bureaucracy; services supported by social health insurance companies; support for GP group practices; payment of GPs; free choice of GP; superordinate quality management; regulations for GP practices
**7. Non-clinical/administrative staff** Subthemes: manner of dealing with patients; competency

### Access

In this section, we discuss the two categories that are related to access to the General Practitioner: *organization and time management of consultation as well as access, availability, and opening-hours of the GP.* Qualitative content analysis of the interviews showed that *organization and time management around the consultation* were found to be very important for participants, in particular with respect to the *“waiting time and circumstances before the consultation.”* Respondents noticed the importance of appointment allocation. It was mentioned positively when they had different options of “*appointment booking”* including the option of coming in without an appointment with a longer waiting time.

*“I can go see my GP anytime, but I know I will have to wait 3-4 hours if I go there without an appointment. And then [with the waiting time in the waiting room] my health would even get worse sometimes. For example, last time I was there because my arm was sore, I waited 3 hours. And a week later I was there again with the flu because I got infected in the GP practice.” (Participant 31, woman, 18-30 years)*

Another important factor was the importance of the “*duration of a consultation.” Many participants felt that GPs did not have enough time to consult them properly; however many added that they felt it was probably due to having to see too many patients. One crucial factor for choosing a specific GP or group practice were opening hours as they should fit to the work schedule of a patient. It was mentioned very often that the opening hours were not manageable with their work schedule and even more so because the waiting times upon arrival were often very long. In addition, easy access was also mentioned quite often; the closer the GP is to home the better.*

*“It was impossible to make an appointment with her. She had certain opening hours when you should go. Often there were at least 20 patients waiting upon arrival.” (Participant 38, woman, 31-45 years)*

### Holistic care/consultation

In this section, we discuss the *two categories* of *professional and human aspects of the consultation*. Both aspects seem to be of high importance to patients, especially in combination. Regarding the *professional aspects of the consultation* participants mentioned several aspects that were important to them, e.g. “*diagnostic and therapeutic skills*” or “*communication skills*” that the GP shows when talking to a patient:

*The doctor asks: “I will prescribe you 400 mg antibiotics is that okay for you?”. She only prescribes it if she knows that I will take them. She will not prescribe it if I do not want to take antibiotics. I can talk to her (GP) about it, we do not have communication problems. (Participant 34, woman, 18-30 years)*

However, prescribing medications was an aspect frequently mentioned in a negative way also, in particular when it was combined with perceived short consultation time. Participants felt that sometimes GPs prescribed a medication without explaining properly to the patient why they prescribed it, and what the medication was supposed to treat.

*“It irritated me when my neighbour told me that she (GP) had prescribed antibiotics but the neighbour did not tolerate it well. And apparently it was a viral thing, how can antibiotics help there?” (Interview 32, woman, 46-60 years)*

When it comes to the “*coordination function”* of GPs regarding patient navigation through the healthcare system, participants desired improvement of this role, as they vocalized being increasingly overwhelmed with the complex pathways of the healthcare system.

*“I had to organize everything alone. Thank God I did not need a doctor coming at home because he would not have come anyways. And when it is very urgent and I really need something immediately I better call the ambulance right away instead of the GP”* (Interview 12, man, 76+)

The person in the quote above had several serious illnesses including cancer and talked about going to the GP only to get drug prescriptions because the GP could not help with other aspects.

Three points mentioned when talking about “*broadening of services or additional services”* were the desire for improvement of preventive services and for additional services such as psychologists, physiotherapists, dieticians, occupational therapists under the same roof, and for alternative treatments such as physiotherapy.

*“I think that the additional services do not have to be provided by general practitioners themselves. But GPs should know about alternative therapies, beside prescribing medication, about physiotherapy, […]. He should know about massage techniques, and, thus, offer alternative treatments.” (Participant 2, man, 31-45 years)*

The category *human aspects of consultation* was considered a very important cornerstone of a successful consultation, in particular, the factors *“established relationship,” “empathy of the GP,” and the “GP´s interest in the living conditions.” Many mention that the most important is the care of physical aspects of health but that psychological aspects also matter to them. People mentioned it as positive when they felt they had been able to build a trusted relationship with their GP. More important than a long-lasting relationship was the aspect of empathy that many people mentioned; when a GP took time to listen to the complaints and problems of the patient taking the larger context of the patient into account.*

*“That’s why these things are very important, to have someone who is calm and friendly, who not only pretends but really cares about me, who not only prescribes an antibiotic and sends me away, but listens to my complaints and tries to address me to understand.” (Participant 34, woman, 18-30 years)*

### Infrastructure and hygiene of the GP practice

The infrastructure of the GP practice, in particular the waiting room mattered to patients. It mattered to the patients if the chairs were comfortable, they often noted whether there were children´s toys and whether they felt the furniture was new and comfortable. One thing some people also noted was an adequate ventilation system as it added to the feeling of being protected against other illnesses in the waiting room. Another factor was whether they felt there was enough space for people in the waiting room. Some respondents mentioned that privacy was important for them, meaning that it was seen as positive when the reception desk was separated from the waiting room where the other patients could possibly hear the patient at the desk.

“*Yes, the waiting room is very big. It’s ventilated and clean, although a lot of people are waiting there. There is modern equipment and it is also good to see that the hygiene is taken care of. It makes it easier to wait.” (Participant 41, man, 18-30 years)*

*“The waiting room of my current general practitioner is bigger, but more minimalistic. So what I found negative once, a few weeks after the birth, was that the armchairs are very hard. But there are also magazines and toys for children.” (Participant 23, woman, 18-30)*

### Healthcare system factors

The category *healthcare system factors* resulting from the question what do you think of the primary care system and what would you change if you could gave many interesting insights. Participants mentioned quite detailed measures on general measures of the healthcare system and on how to improve the situation at the practice level. For example, social health insurance companies were identified for improvement. That included bureaucratic barriers, electronic prescriptions, referrals, and sick leave certificates. Moreover, GPs should be paid differently—not according to the number of patients but a system that takes into account the quality of care; and group practices should be enabled and supported.

*“I think that from an organizational point of view it would be important to pay medical services in a different way. Not only – medical consultation, paying fee-for-service. Instead of that financing should simply work differently. Mass of patients in the […] shouldn’t be necessary to finance good medical practice. Instead of that quality should be rewarded because like this, patients receive better treatment.” (Participant 3, woman, 31-45 years)*

### Non-clinical/administrative staff

The non-clinical/administrative staff of the GP practice is mentioned several times during the interviews. Often the contact that a patient has with the assistant can be crucial especially in GP practices with social insurance contracts, as here the GP usually does not have more than a few minutes by patient. Therefore, the assistant plays a crucial role in building trust, continuity and confidence for the patient.

*“(...) his secretary - she is very friendly, very good. In my opinion, this is very important, even more so than for the doctor. Of course, having a good doctor is also important, but it’s more important to have a friendly secretary, who takes care of you.” (Participant 35, woman, 18-30 years)*

## Discussion

When patients in Austria are given a voice about their perceptions and desires regarding their healthcare by GPs, they talk about seven aspects in particular. These are coordination and time management around consultation, access to care including availability and opening hours, professional and human aspects of the consultation, infrastructure, and hygiene of the waiting room, general healthcare system factors, and non-clinical/administrative staff (Table 2).

It is not surprising that the availability of primary care services within their vicinity as well as adequate opening hours are important factors for the participants, as these are foundational elements to access to care [5, 6, 8]. Adequate opening hours are particularly important for those individuals whose employment limits the opportunity for seeking daytime care. Morgan and colleagues showed that satisfaction with opening-hours improved slightly for practices offering extra appointments, for example on Saturdays [22]. Additionally, an extension of the daily coverage of primary care services might reduce inappropriate admissions to emergency services [23].

Most important for the participants, however, is the category of organization and time management around consultation and, in particular, the waiting time before the consultation in the waiting room. Previous publications have shown that waiting time is heavily associated with satisfaction with the service, as well as willingness to return [24–26]. Another important factor found in this study regarding time management is the length of the consultation itself. Previous publications have shown that time spent with the physician was the strongest predictor of patient satisfaction [24]. In addition, this factor is closely associated with waiting time. “*The decrement in satisfaction associated with long waiting times is substantially reduced with increased time spent with the physician [...]”* [24]. The consultation time in GP practices in Austria is approximately 5 min, which is in stark contrast to for example Swedish GPs who have a mean time of 22 min per patient [27]. Five minutes is a remarkably short period to address all the desired professional and human factors within the consultation that are relevant for the study participants. In addition, consultation length has implications for patient safety, management of chronic disease, diagnoses, and outcomes [27, 28].

One study by Odgen *et al*. shows that the majority of patients might underestimate consultation length. In this study a preference for more time was correlated with a dissatisfaction with the emotional (human) aspects of the consultation but not with the information and examination components of the consultation [29]. This shows the relevance of human factors for forming a successful doctor–patient relationship. The doctor–patient relationship influences patients’ compliance with treatment and is an important cornerstone for continuity of care. *“Satisfaction with the doctor–patient relationship is a critical factor in people’s decisions to join and stay with a specific organization* [30]*.“* In Austria, these factors persist, and this is in part due to the free choice of GPs, an element of the system that must be maintained [31]. In our study, many participants were concerned with prescriptions of medicine that they judged too fast or too high a quantity or dosage. They felt that often the physician would prescribe a drug without further explanation. Another desire expressed in this study was to expand or supplement the existing services by including supplementary primary care professions into the GP practice. This desire is echoed by international publications demonstrating methods to improve patient care [32–34].

In addition, participants of this study urgently called for a better coordination and navigation function between the level of care and within primary care. As described above in detail, in Austria, first contact and coordination with primary care are not required to be able to access the secondary level of care (medical specialists and hospitals) [10, 11]. In contrast, “*[a] strongly developed primary care sector, as defined by the expert panel on effective ways of investing in health (EXPH), commissioned by the European Commission, should ‘play a central role in the overall coordination and continuity of people’s care’* [32]. Such a coordination and navigation role could protect patients from unnecessary harm due to getting lost in the complex healthcare system, avoiding unnecessary and multiple diagnostics, unnecessary hospital admissions, or wrong treatment strategies [9, 32–34].

### What are the implications for primary care reform and reform in Austria?

Fortunately, several aspects of Austrian healthcare reform goals align with the perceptions and desires expressed by patients in this study. These include expanded office hours, expanded and enhanced service profiles, integration of an interdisciplinary team into practices, and support of group practices and primary care centres. In addition, free choice of GPs and universal health coverage should be maintained.

However, other important factors for patients’ satisfaction and willingness to access and return to a service need improvement, according to our findings. These are organization and time management around consultation, consultation length, and the coordination function of primary care. In this study, overarching system factors were additionally identified which align with a study based in the USA:

“[R]*espondents were quick to note that existing fee-for-service payment does not reimburse care coordination efforts. Because there are no payments for such activities as following up on referrals or communicating with patients outside of the office, physicians do so at the expense of other, billable activities”* [35].

A primarily fee-for-service payment scheme, which is the case for Austria, correlates strongly with more patients, less time per patient, and longer waiting times in a practice. This is in contrast to value- and need-based payment schemes [36]. Most participants were aware that their suggestions for improving primary care generally lie outside of the hands of the GPs themselves. Rather, the larger systemic factors, policies, and priorities of health insurance companies drive much of the way care is accessed and delivered. Interestingly, those factors acknowledged by the participants of this study were also identified as critical by Kringos in 2012 and have been recommended at the international level for comprehensive and effective primary care [8, 9, 32, 33].

## Strengths and limitations

One strength of this study is the fact that it is the first of its kind in Austria on patient’s desires for improvement of primary care in Austria. Another strength is the approach, employing an exploratory qualitative study design. The sample of participants is both a strength and a limitation. With 41 participants from different age groups, sex, counties, and language backgrounds, data saturation could easily be reached for this sample. However, with regard to age and educational level, more individuals with higher educational levels from the age group 46 until 60 years of age were included. Additionally, no participants from the more rural southern and western parts of Austria were recruited, and therefore there are potentially missing aspects from these regions. As this is a qualitative study we cannot generalize the findings which is why we suggest that more qualitative and quantitative studies are needed in order to adequately take patients opinions into account for further steps in healthcare reform. Furthermore, issues such as memory bias, recall bias, or bias of possible social desirability could have had an impact on the reports of interviewees in this study. As a last limitation, it must be mentioned that the snowball sampling method bears the risk of a community bias, where the first contact has a dominant impact on the sample and represents a potentially limited network. However, as four students carried out the interviews that bias could be limited. The fact that we conducted interviews in German and Turkish is a strength as it takes into account that German is not the first language of much of Austria´s population today. The potential limitation in that regard is that there might be deviations in meanings of interview content due to the two rounds of translation—from Turkish to German and from German to English. However—as it was the person who did the interviews who translated them to German, the student could be attentive to make sure that the original meaning was kept. Last but not least it is important to address that interviews are from 2018. The data collection by the students did not take place simultaneously, but gradually, whenever students became interested in this research question. Data collection took an average of one year per federal state/language. By the time all students had finished, however, 5 years had passed and data analysis took another 2 years due to funding. Then the pandemic began and the first author, who also works as a doctor, had no team and no time to push ahead with the publication until SW joined the team in the summer of 2023. However, the situation in primary care in Austria has not changed significantly in the last eight years which is why the results are still relevant to date.

## Conclusion

Appreciating and acknowledging the perception of patients provides a deeper context and perspective into primary healthcare access and delivery. This study added new aspects like the desire for a coordination and navigation function for general practice, inter-professional care teams as well as improved time aspects around the consultation. Time and coordination aspects of primary care are about to be improved, while structural changes remain necessary for full system reform and improvement. For the next steps of reform patients’ voices should be integrated on a regular basis to evaluate the success and the achievement of the objectives of reform.

## Supplementary data

Supplementary material is available at *Family Practice* online.

cmae031_suppl_Supplementary_Materials

## Data Availability

The data used for this article will be made available upon request to the first or corresponding author.
